# Clinical Implications of Sickle Cell Thalassemia on Pregnancy Outcomes: A Systematic Review

**DOI:** 10.3390/diagnostics16182933

**Published:** 2026-09-11

**Authors:** Angeliki Gerede, Sofoklis Stavros, Anastasios Potiris, Efthymios Oikonomou, Chrysi Christodoulaki, Aikaterini-Lydia Vogiatzoglou, Christos Chatzakis, Ekaterini Domali, Nikoletta Koutlaki, Makarios Eleftheriadis

**Affiliations:** 1 Department of Obstetrics and Gynecology, Democritus University of Thrace, 691 00 Alexandroupolis, Greece; eftoikonomou@outlook.com (E.O.); nkoutlak@med.duth.gr (N.K.); 2 Third Department of Obstetrics and Gynecology, Medical School, National and Kapodistrian University of Athens, University General Hospital “ATTIKON”, 124 62 Athens, Greece; apotiris@med.uoa.gr (A.P.); vogiatzoglou.lydia@gmail.com (A.-L.V.); 3 Department of Obstetrics and Gynecology, Chania General Hospital “St. George”, 733 00 Chania, Greece; christodoulakichr@hotmail.com; 4 Second Department of Obstetrics and Gynecology, Medical School, National and Kapodistrian University of Athens, “Aretaieion” University Hospital, 115 28 Athens, Greece; cchatzakis@gmail.com (C.C.); melefth@med.uoa.gr (M.E.); 5 First Department of Obstetrics and Gynecology, Medical School, National and Kapodistrian University of Athens, Alexandra Hospital, 115 28 Athens, Greece; kdomali@yahoo.fr

**Keywords:** sickle cell thalassemia, sickle cell disease, sickle cell anemia, sickle cell trait, HbS/beta-thalassemia, pregnancy outcomes, maternal complications, fetal outcomes, neonatal outcomes, preeclampsia, preterm birth, multidisciplinary care

## Abstract

**Background/Objectives**: Sickle cell thalassemia (HbSβ-thal) and related sickle cell disease (SCD) genotypes are associated with substantial maternal, fetal, and neonatal morbidity during pregnancy. This systematic review aimed to summarize pregnancy outcomes and clinical management strategies in women with HbSβ-thal and related hemoglobinopathies, with an additional narrative comparison to sickle cell trait (SCT)—a distinct, heterozygous, and generally benign carrier state that is not a form of SCD. **Methods**: A systematic literature search was conducted in PubMed, MEDLINE, Web of Science, EMBASE, and Scopus for studies published between 2016 and 2026 using terms related to sickle cell anemia, sickle cell disease, sickle cell thalassemia, HbS/beta-thalassemia, sickle cell trait, pregnancy outcomes, maternal complications, neonatal outcomes, fetal outcomes, and clinical/obstetric management. Studies reporting maternal, fetal, or neonatal outcomes were included, while letters, commentaries, conference abstracts, and presentations were excluded. Study selection followed PRISMA principles. Risk of bias was assessed using appropriate tools according to study design, including the Newcastle–Ottawa Scale (NOS), AMSTAR 2 (A MeaSurement Tool to Assess systematic Reviews), SANRA (Scale for the Assessment of Narrative Review Articles), and CARE (CAse REport) guidelines. SCT terms were included in the same search strategy, so that SCT studies were retrieved and screened through the same PRISMA process and are reported as a prespecified comparator subgroup. **Results**: Fourteen studies met the inclusion criteria: ten reporting outcomes in HbSβ-thal and related SCD genotypes and four reporting outcomes in SCT. The most frequently reported maternal complications were severe anemia, vaso-occlusive crises (VOCs), preeclampsia, thromboembolic events, infections, acute chest syndrome, cesarean delivery, and postpartum complications, which led to a significant increase in hospitalization rates. Fetal and neonatal outcomes were also unfavorable, with increased rates of preterm birth, low birth weight, intrauterine growth restriction, placental insufficiency, and neonatal intensive care admission. Placental vascular malperfusion and low maternal hemoglobin were important mechanisms underlying fetal growth restriction. By contrast, the included SCT literature suggested outcomes generally closer to the general obstetric population, with a smaller number of specific associations (e.g., preeclampsia, intrauterine fetal death) reported inconsistently across studies. **Conclusions**: Pregnancies complicated by HbSβ-thal and related hemoglobinopathies remain high-risk and require early identification, preconception counseling, multidisciplinary care, close antenatal surveillance, individualized transfusion strategies, and postpartum thromboprophylaxis when indicated. Prospective genotype-specific studies, particularly isolating HbSβ-thal from broader SCD cohorts, are needed to optimize evidence-based management.

## 1. Introduction

The term “sickle cell disease” (SCD) refers to an inherited disorder of hemoglobin characterized by a mutation in one nucleotide of the beta-globin gene, yielding abnormal hemoglobin (HbS). Three genotypes are included in the SCD category: hemoglobin SS (HbSS), hemoglobin SC (HbSC), and hemoglobin S-beta-thalassemia (HbS-beta-thal). Among these, HbSS is the most serious form, which is defined by chronic hemolytic anemia, repeated VOCs, progressive organ damage, and a short life span. As for the HbSC disease, it typically follows a milder clinical course, where complications can still occur. In HbS-beta-thalassemia, two types can be distinguished: HbS-beta(0)-thalassemia, which is functionally similar to HbSS due to the absence of normal beta-globin production, and the generally milder HbS-beta (+)-thalassemia, in which some hemoglobin A (HbA) is still produced. On the other hand, sickle cell trait (SCT) is defined as a heterozygous carrier state, which indicates that individual carriers of the trait do not usually show any symptoms in ordinary conditions. The difference between SCD (including HbSβ-thal) and SCT is significant since pregnancy in these two cases can lead to different outcomes. Specifically, women with SCD have an elevated risk of maternal and perinatal complications, whereas women with SCT generally have outcomes closer to the general population, with only rare adverse events reported [1,2,3,4].

Pregnancy is regarded as a high-risk period for women with SCD due to physiological alterations, including increased oxygen consumption, a change in blood flow, hemodilution, and increased coagulation, which exacerbate the underlying pathophysiology of the disease. These changes result in increased SCD crises, specifically increasing the frequency of VOCs, and predispose women to infections, thromboembolic events, and organ failure [1,2,3,4].

Maternal complications reported in the literature include anemia, acute chest syndrome, preeclampsia, and urinary tract infections, with maternal death rates being higher than in the general pregnant population. Whereas most fetuses that grow and develop inside a mother’s womb will likely be born prematurely, have a low birth weight (LBW), or grow slowly due to inadequate oxygen and placental function on the mother’s part during pregnancy [4].

The broader category of SCD, HbS-beta-thalassemia, is an important but understudied genotype. Although some studies have reported pregnancy outcomes by SCD genotype, the maternal and fetal risks associated with HbS-beta-thalassemia remain incompletely defined [5,6]. Therefore, risks must be accurately identified and monitored at every stage of a mother’s pregnancy to optimize outcomes [3,4]. The purpose of this review is to investigate the outcomes in women with HbS-beta-thalassemia, with a particular focus on maternal and fetal complications, as well as the different types of clinical care methods that may provide mothers and their babies with the greatest opportunity to achieve optimal pregnancy outcomes and additionally situates these findings against the comparatively sparse literature on SCT in pregnancy.

## 2. Materials and Methods

A comprehensive literature search was performed across five databases: PubMed, MEDLINE (Ovid), EMBASE (Ovid), Scopus, and Web of Science Core Collection (last searched 31 July 2026). The strategy combined database-specific controlled vocabulary (MESH terms in PUBMED and MEDLINE; EMTREE terms in EMBASE) with free-text terms searched in title, abstract and keyword fields, using truncation and wildcards wherever the interface permitted. Two concepts were combined with the Boolean operator AND: (i) a haemoglobinopathy concept (“sickle cell disease”, “sickle cell anaemia/anemia”, “sickle cell thalassaemia/thalassemia”, “HbS/beta-thalassaemia”, “haemoglobin S”, “sickle cell trait”, “sickle trait”, HbAS, “haemoglobinopathy”) and (ii) a pregnancy concept (“pregnancy”, “pregnant”, “obstetric”, “maternal”, “antenatal”, “perinatal”, “peripartum”, “postpartum”, “fetal/foetal”, “neonatal”, “birth outcome”), with terms within each concept combined using OR. The following limits were applied in every database: English language; human studies; and publication date from 1 January 2016 to 30 July 2026. Editorials, letters, comments, notes, and conference abstracts or proceedings papers were removed using the publication-type or document-type filter of each interface. No limits were applied for study design, geographical region, sample size or study setting. The basic architecture of all databases remained intact, whereas the search algorithm was modified for each database’s syntax. This review includes observational and internationally published studies on complications and outcomes among pregnant women. The methodology for this systematic review was developed and executed following the guiding principles of the International Prospective Register of Systematic Reviews (PROSPERO) and reported in accordance with the PRISMA guidelines. However, a formal review protocol was not prospectively registered. The strategy was peer-checked against the PRESS 2015 checklist. In addition to the database searches, the reference lists of all included articles and of relevant reviews were hand-searched for further eligible studies.

**Figure 1:**
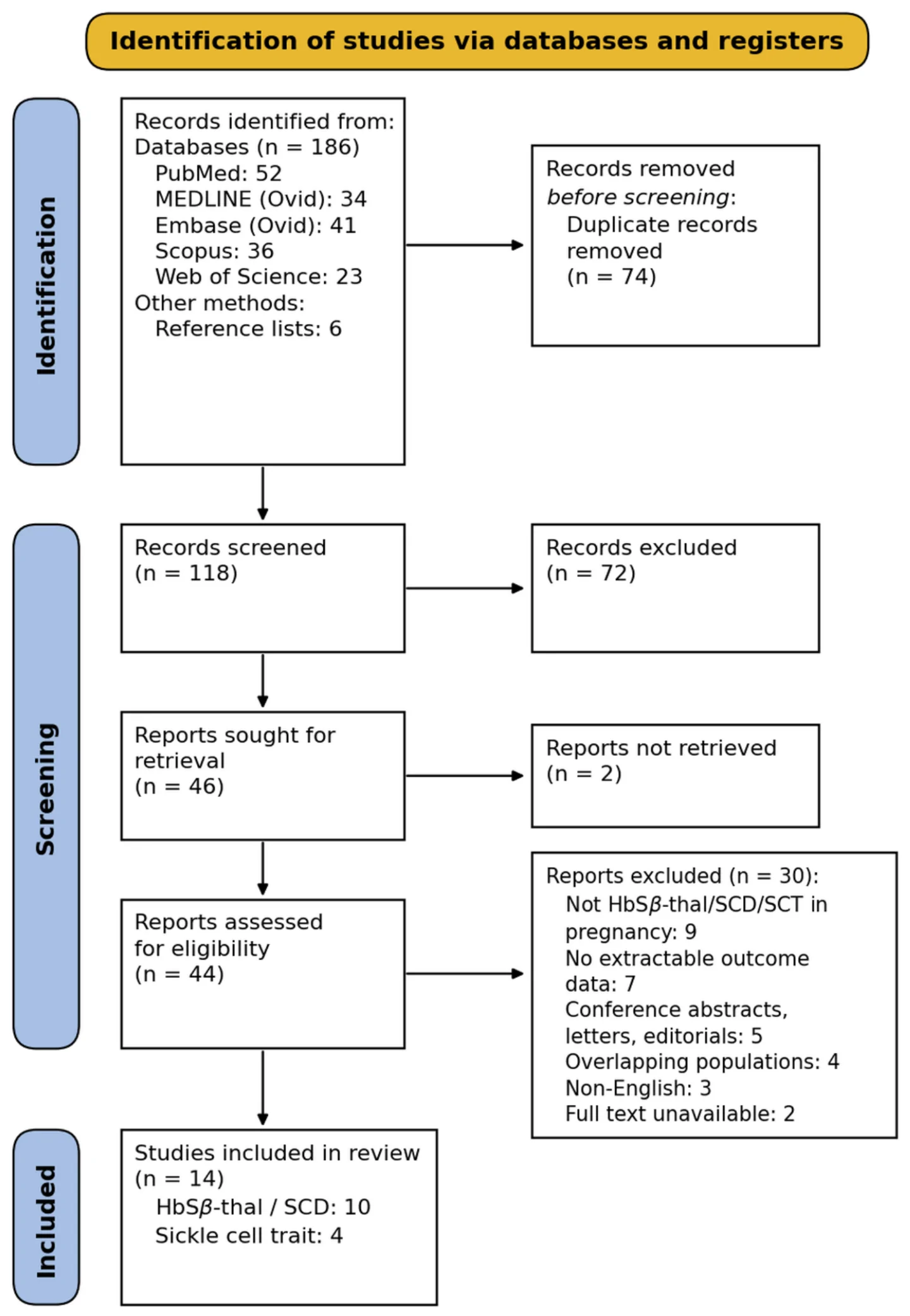
A PRISMA flow diagram of the selection process for the included studies.

Initially, duplicates were detected through a search of the identical records database. Two independent reviewers (A.P. and E.O.) then reviewed the articles identified through a title and abstract review, wherein, for each of the studies included, the data was collected and categorized based on the following outcomes: publication date, study design, and the maternal/fetal complications of sickle cell anemia and HbSβ-thal reported in pregnancy, including severe anemia, VOC, preeclampsia, LBW, and intrauterine growth restriction (IUGR). The selection procedure is shown in Figure 1. The search retrieved 186 records (PubMed, *n* = 52; MEDLINE via Ovid, *n* = 34; Embase via Ovid, *n* = 41; Scopus, *n* = 36; Web of Science Core Collection, *n* = 23), and a further 6 records were identified by hand-searching reference lists. After 74 duplicate records were removed, 118 records were screened by title and abstract, of which 72 were excluded. Full texts were sought for 46 reports; 2 could not be retrieved, and 44 were assessed for eligibility. Of these, 30 were excluded (not addressing HbSβ-thal, SCD or SCT in pregnancy, *n* = 9; no extractable maternal, fetal or neonatal outcome data, *n* = 7; conference abstracts, letters or editorials, *n* = 5; overlapping or duplicate study populations, *n* = 4; non-English publications, *n* = 3; full text unavailable, *n* = 2). Ultimately, 14 studies met the inclusion criteria: 10 reporting outcomes in HbSβ-thal and related SCD genotypes and 4 reporting outcomes in SCT. SCT terms were included in the search string executed in every database; the four SCT studies identified were screened, selected and quality-appraised using the same procedure and the same appraisal tools as the HbSβ-thal/SCD studies (Table 1), and are reported as a prespecified comparator subgroup of the same evidence base.

**Table 1:** Summary of the included studies: clinical characteristics and risk-of-bias/quality appraisal.

#	First Author (Year)	Country/Setting	Study Design	Sample Size	Main Outcomes	Assessment Tool	Score/Rating	Overall Risk of Bias	Methodological Limitations/Notes
[1]	Figueira et al. (2022)	Brazil	Literature review (secondary literature)	N/A (narrative synthesis)	High rates of anemia and preterm delivery	SANRA	10/12 Points	Low-Moderate	Relies on retrospective chart reviews; potential selection bias. Several figures are secondary citations within this review rather than its own primary data.
[2]	Shegekar & Pajai (2023)	India	Narrative review (secondary literature)	N/A (narrative synthesis)	Importance of screening and multidisciplinary care	SANRA	8/12 Points	Moderate	Lacks a systematic literature search strategy; susceptible to selection bias.
[3]	Sunanda & Impana (2023)	India	Case report (primary, single-patient)	*N* = 1	Successful multidisciplinary management with term delivery	CARE	11/13 Items	High (inherent)	High risk of publication bias; lacks generalizability.
[4]	Oteng-Ntim et al. (2021)	United Kingdom	Clinical guideline/evidence review (secondary literature)	N/A (evidence synthesis)	Increased rates of preterm birth, low birth weight, and maternal complications in SCD	AMSTAR 2	High Confidence	Low	Robust methodology; strictly adheres to British Society for Hematology standards.
[5]	Virot et al. (2022)	France	Multicenter observational study (primary)	37 women; 56 pregnancies	Increased maternal and neonatal complications reported	NOS	8/9 Stars	Low	Strong multicenter design; well-defined outcomes for transfused β-thalassemia.
[6]	Habibi et al. (2023)	France (multicenter; incl. Greece)	Prospective cohort study (ESCORT-HU) (primary)	128 pregnancies	High maternal mortality and adverse neonatal outcomes	NOS	8/9 Stars	Low	Excellent prospective tracking (ESCORT-HU); robust control of confounders.
[7]	Elenga et al. (2016)	French Guiana (France)	Observational study (primary)	44 women; 62 pregnancies	High-risk pregnancies with increased maternal complications	NOS	6/9 Stars	Moderate	Limited sample size; lacks a perfectly matched healthy control group.
[8]	Asare et al. (2019)	Ghana	Cohort study (primary)	Not reported in source abstract	Placental dysfunction is associated with fetal IUGR	NOS	7/9 Stars	Low-Moderate	Clear outcome definitions for acute chest syndrome (ACS); limited follow-up duration.
[9]	Dora et al. (2019)	India	Prospective comparative study (primary)	178 women (59 SCD; 119 sickle cell trait)	Increased painful crises and adverse maternal-fetal outcomes in SCD vs. trait	NOS	7/9 Stars	Low-Moderate	Good comparison between disease and trait women; single-center limitations.
[10]	Corsia et al. (2025)	France (nationwide)	Nationwide cohort study (primary)	1022 SCD births (of 5,752,080 total pregnancies)	Increased maternal and perinatal complications in pregnancies complicated by SCD	NOS	9/9 Stars	Low	Exceptional statistical power (nationwide data); excellent comparability and outcome ascertainment.
[11]	Wellenstein et al. (2019)	USA (Kaiser Permanente Northern California)	Retrospective cohort study, SCT (primary)	31,840 women (868 with SCT)	Higher rate of pregnancy-induced hypertension (15.6% vs. 12.2%); SCT not an independent predictor after adjustment for ethnicity	NOS	8/9 Stars	Low	Large integrated healthcare cohort with multivariable adjustment; retrospective design; single healthcare system.
[12]	Hulsizer et al. (2023)	United Kingdom (UK Biobank)	Population-based cohort study, SCT (primary)	Self-reported Black UK Biobank participants (*n* not stated in source abstract)	SCT associated with increased risk of preeclampsia (RR 2.39, 95% CI 1.09–5.23), bacteriuria/UTI and pregnancy loss	NOS	8/9 Stars	Low	Large population-based sampling with genotype-defined exposure; outcome ascertainment from linked records; restricted to UK Biobank participants.
[13]	Buhusayyen et al. (2022)	Bahrain	Retrospective cross-sectional case–control study, SCT (primary)	934 women (460 SCT; 474 controls)	Higher intrauterine fetal death (3% vs. 0.2%) and cesarean delivery (28.7% vs. 21.7%); no difference in hypertensive disorders, gestational diabetes, small-for-gestational-age (SGA) infants, or preterm delivery	NOS	6/9 Stars	Moderate	Single-center, single-year record review; limited adjustment for confounders; outcome definitions not externally validated.
[14]	Ali et al. (2023)	Sudan	Prospective cohort study, SCT (primary)	94 women (34 SCT; 60 controls)	Maternal and neonatal anemia, LBW, and increased miscarriage, stillbirth and NICU admission	NOS	7/9 Stars	Low-Moderate	Prospective recruitment with serial hematological measurement; small sample size; single center.

NOS: Newcastle–Ottawa Scale; AMSTAR 2: A Measurement Tool to Assess Systematic Reviews; SANRA: Scale for the Assessment of Narrative Review Articles; CARE: CAse REport guidelines; N/A: Not Applicable/Not Available.

The studies included in this review examined maternal, fetal and neonatal outcomes in pregnancies with SCD, with particular focus on sickle cell thalassemia (HbS/beta-thalassemia). The most frequently occurring maternal complications were reported as serious anemia, VOCs, preeclampsia, thromboembolic events, and increased rates of hospitalization [1]. Several studies also contained information regarding negative fetal and neonatal outcomes, including preterm birth, LBW, IUGR, and placental insufficiency. Concerning evidence duplication, it should be reported that of the fourteen studies in Table 1, eleven are primary studies contributing original patient-level data [3,5,6,7,8,9,10,11,12,13,14], while three are secondary syntheses retained for narrative/quantitative context: two narrative literature reviews [1,2] and a clinical practice guideline incorporating a structured evidence review [4]. Four of the included studies [11,12,13,14] report pregnancy outcomes in women with SCT; these were retrieved by the same search and are summarized alongside the HbSβ-thal/SCD studies in Table 1.

Two reviewers (A.G. and S.S.) independently conducted a formal quality and risk of bias evaluation to ensure the methodological rigor of the included literature. Because of the variability of the types of studies included, different validated appraisal tools were used: (a) Observational and cohort studies [5,6,7,8,9,10,11,12,13,14] were assessed using the Newcastle–Ottawa Scale (NOS), which awards up to 9 stars based on the selection of the study groups, the comparability of the groups, and the ascertainment of either the exposure or outcome of interest. Studies scoring 7 or more were deemed to be of excellent quality (low risk of bias). (b) The clinical guideline [4], which incorporates a structured evidence review, was appraised using AMSTAR 2 (A Measurement Tool to Assess Systematic Reviews) criteria to evaluate the adequacy of the literature search, the risk of bias in primary studies, and the suitability of meta-analytical methods. (c) SANRA (Scale for the Assessment of Narrative Review Articles) framework was used to assess narrative reviews [2], including justification of the article’s importance, description of the literature search, and scientific reasoning. (d) Case Report [3] was examined according to the CARE (CAse REport) guidelines checklist to guarantee the transparency and completeness of the clinical narrative, diagnostic findings, and treatment interventions. Disagreements between reviewers were addressed by consensus or by a third reviewer (A-L.V).

The methodological quality of the fourteen included studies varied depending on the study design. The cohort and observational studies generally demonstrated moderate-to-high quality, with most scoring between 6 and 8 on the NOS. The most frequent methodological limitation among the observational cohorts was the lack of adequate control groups representing the general obstetric population and a high risk of confounding bias due to retrospective data collection. The included guideline/evidence review demonstrated high methodological rigor, adequately registering protocols and performing robust literature sweeps (AMSTAR 2 rating: High/Moderate). The included case report successfully adhered to CARE guidelines, though its inherent design limits its generalizability. The four SCT studies were appraised with the same NOS instrument and scored between 6 and 8 stars, with the principal limitations being retrospective record review and, in two studies, single-center recruitment (Table 1). A summary of clinical characteristics and the risk of bias assessment is detailed in Table 1.

## 3. Results

### 3.1. Maternal Complications

#### 3.1.1. Vaso-Occlusive Crises (VOCs)

VOCs are one of the major complications of SCD during pregnancy. According to a report, the occurrence of VOCs in pregnant women suffering from SCD is about 28.8%, compared to 4.2% in pregnant women suffering from SCT [1]. Among women with HbSβ-thalassemia, frequent episodes of severe anemia and recurrent VOCs that cause acute pain in the bone or joint are the most common reasons that women are hospitalized while pregnant [1]. It is also reported that there are higher rates of complications in pregnancies with SCD compared to unaffected pregnancies [7], requiring increased blood transfusion and higher rates of antenatal hospitalization.

#### 3.1.2. Preeclampsia

One of the most critical effects of preeclampsia on maternal health can be severe and result in long-term complications for both mother and child. The following are examples of mechanisms linked to high blood pressure in women diagnosed with preeclampsia: chronic vascular dysfunction, increased inflammation, oxidative damage, and decreased blood supply to the placenta [2,10]. Based on the available literature, a woman with SCD or related disorders has an over five-fold higher risk of a preeclamptic episode than the general obstetric population [10]. A French study reported that pregnant females with SCD had a prevalence rate of 9.6% versus 1.7% in the general obstetric population [11]. The incidence of hypertension and preeclampsia also seems to be higher among women with SCT. According to a population-based cohort study derived from the UK, SCT carriers are at a higher risk of developing preeclampsia [12]. A large retrospective analysis from Kaiser Permanente similarly reported a higher prevalence of pregnancy-induced hypertension among SCT women (15.6% compared to 12.2%; *p* = 0.003) [11]. However, in multivariable analyses controlled for ethnicity, SCT was not an independent predictor of hypertensive disorders, indicating that confounding factors may be responsible due to association [11]. Notably, the British Society for Hematology advises that women with SCT need to be monitored for the development of hypertensive complications during pregnancy [4]. It is important to identify signs of hypertension early on so that closer monitoring can be completed throughout the pregnancy due to the presence of chronic maternal hypoxia and decreased placental perfusion of SCD [3].

#### 3.1.3. Thromboembolic Events

Thromboembolic events are another major concern. A systematic review determined that chronic injury to endothelial cells, inflammation, and hypercoagulability, mainly caused by pregnancy and SCD, contribute to the increased incidence of venous thromboembolism [15]. The same coagulation cascade has also been proposed to contribute to placental thrombosis and impaired uteroplacental perfusion, although this specific pathway is less directly studied. The French cohort study reported a pulmonary embolism prevalence of 0.7% in women with SCD, while the control study found an incidence of 0.02% [10]. However, a substantial study on 12,000 pregnant women suggested there is no significant difference in the peripartum incidence of venous thromboembolism (0.44% vs. 0.49%) between the SCT carriers and women with normal hemoglobin, even though SCT has been related to an increase in incidence of venous thromboembolism among the non-pregnant women [16]. Previous studies and guidelines implement prevention strategies for thrombosis in high-risk conditions associated with sickle cell disease and hemoglobinopathies [3,4].

#### 3.1.4. Acute Chest Syndrome and Postpartum Outcomes

Acute chest syndrome is a major complication that can be a fatal pulmonary complication. It can occur in up to 10% of pregnant women with SCD [8,17,18]. It can typically show symptoms such as fever, breathing issues, and new areas of lung infection, leading to death. Acute chest syndrome is the number one cause of death for adult patients with SCD [8]. Whereas in patients with SCT the acute chest pain is rare and occurs under physiological stress conditions during pregnancy [19]. More often, infections are seen, with urinary tract infections (UTIs) being the most common type of disease seen during pregnancy [17,18]. There is also one study reporting that the SCT carriers have an elevated risk of UTIs and bacteriuria [12]. The French study indicated that genitourinary infections were more common in women with SCD, at 8.4%, compared to only 5% of the controls. Women with SCD had a greater risk of developing postpartum hemorrhages (8.3% vs. 4.1%) and had higher rates of cesarean section (52.8% vs. 18.2%) [10]. Importantly, from the SCT data, it was found that pregnant women with SCT had a significantly greater prevalence of intrauterine fetal death (3%, *p* < 0.001). However, regarding other variables measured, such as hypertensive disorders, gestational diabetes, SGA, and preterm delivery, there were no statistically significant differences between the two groups [13]. Similarly, SCT pregnancies in another study from a prospective cohort were shown to be associated with lower birth weights, more miscarriages, stillbirths, and neonatal unit admissions compared to controls [14]. Figueira et al.’s review reports the risk of postpartum complications (hemorrhage, infection, thrombosis) as higher specifically in the HbS-beta-thalassemia group (57%) than in HbSS (18%) or HbSC (13%) groups [1], suggesting this genotype may predispose patients to a higher risk of experiencing postpartum complications [1].

### 3.2. Fetal and Neonatal Outcomes

Fetal and neonatal outcomes in pregnancies complicated by HbSβ-thal and other SCD genotypes are affected by poor uteroplacental circulation, chronic maternal anemia, and the inflammatory state caused by the underlying hemoglobinopathy.

#### 3.2.1. Preterm Birth

Preterm delivery is one of the most common complications of pregnancy. A study found a 30% incidence of preterm delivery across all SCD genotypes, with no differences between groups (HbSS, HbSC, or HbSβ) [1]. Specifically, this incidence suggests that preterm birth risk extends to SCT pregnancies at rates like other SCD genotypes [13]. Similar findings documented that 25.9% of women delivered preterm in an SCD cohort that included 9.6% of women who had HbS-β-thalassemia [20]. There is consensus that the incidence of preterm delivery is elevated among all affected women and infants; for example, two published studies documented preterm birth rates exceeding 25% among diverse populations. Also, the nationwide French cohort study showed that preterm birth rates among women with SCD were 28.5%, compared to only 5.6% for pregnancies not affected by SCD (*p* < 0.001) [10]. In addition to the documented variation in overall incidence rates of preterm delivery, a genotype-specific analysis showed that women with the HbSS genotype delivered preterm at 41%, while those with the HbSC genotype did so at 22% [7]. Among women with β-thalassemia, cohort data indicate that rates of preterm birth in thalassemia major are like those in intermedia, but overall, the evidence remains limited [5].

#### 3.2.2. Low Birth Weight (LBW)

Among women with β-thalassemia, the incidence of delivering small-for-gestational-age (SGA)-born babies is reported to be similar in thalassemia major and intermedia groups, though published cohorts, however, have generally failed to demonstrate differences [5]. Infants with SGA had the greatest risk among mothers with the most prevalent (HbSS) genotype. In a case–control study, the researchers also found that the rates of IUGR and LBW were higher among women with SCD (HbSS) [21]. Furthermore, in most cases (57%), the women with SCD experienced VOCs that resulted in hospitalization prior to delivery.

#### 3.2.3. Intrauterine Growth Restriction

IUGR and LBW are commonly linked to pregnancies affected by SCD. For example, researchers found that 38.89% of SCD pregnancies resulted in IUGR, and 56.94% of neonates were born with LBW [22]. In a study, it was confirmed that the average weight of an infant with SCD at delivery was 2965 g versus 3457 g for a non-SCD infant, and the rate of IUGR was reported to be 10–13%. A meta-analysis was also performed, including nearly 10,000 SCD pregnancies, and it indicated that women with SCD have greater risks for both IUGR and LBW across all SCD genotypes [23]. Genotype differences also exist for infant growth in SCD pregnancies. For instance, while the overall incidence of preterm birth was shown to be 30%, and there were no statistically significant differences among the SCD genotypes, the hemoglobin levels (grams per deciliter) at the first antenatal visit were significantly (*p* < 0.01) lower in HbSS (7.7 g/dL) than in either HbSC (9.7 g/dL) or HbSβ-thalassemia (8.4 g/dL). This could partially explain the more severe growth restriction seen in infants born to mothers with homozygous disease [1].

#### 3.2.4. Placental Insufficiency

Restricted fetal growth is caused by a lack of blood flow through the mother’s placenta since the mother has suffered damage to blood vessels in the placenta and/or due to having chronic anemia. There are also different known types of early placental damage leading to restricted fetal growth. Figueira et al.’s review reports that, of 15 placentas examined from mothers with SCD, 10 showed abnormal findings consistent with maternal vascular malperfusion [1]. Another study by Nwachukwu et al. shows that, of the 48 sickle cell placentas, a large percentage had abnormal findings consistent with maternal blood vessel disease; specifically, 79% were diagnosed with maternal blood vessel disease. The most common abnormal findings were small placentas for gestational age, maternal blood vessel disease (also referred to as decidual vasculopathy), an increased number of syncytial knots, increased fibrin, immature placental villi, thrombus, and placental infarct. In addition, maternal hemoglobin levels of less than 8 g/dL during pregnancy were correlated with a significantly increased incidence of fetal IUGR (47% vs 14%, *p* = 0.0074). These findings support the link between chronic hemolytic anemia, decreased placental blood flow, and fetuses with restricted fetal growth [24].

## 4. Clinical Management During Pregnancy

### 4.1. Preconception Care

Preconception counseling to help prevent pregnancy complications, partner testing, and being healthy are essential for women with sickle cell thalassemia. It is important to stop the use of hydroxyurea (hydroxycarbamide) at least three months before trying to conceive because it may cause birth defects, as well as to prevent iron chelation therapy before conceiving, helping in this way to prevent any possible harm to a woman or her unborn child from iron chelation. All women should be prescribed folic acid (5 mg daily), and prophylactic antibiotics should be used only in women with laboratory-validated evidence of iron deficiency [25]. The baseline iron status assessment must be carefully done for patients with thalassemia, as patients may require aggressive chelation related to the iron overload from past transfusions before pregnancy can be recommended [26].

Several studies emphasize the overall significance of carrier detection and genetic counseling for partners. Effective management of the preconception phase requires a multidisciplinary team (MDT) approach. This is consistent with the Society for Maternal-Fetal Medicine’s recommendation that women with SCD be co-managed by a multidisciplinary team from the beginning of pregnancy [25].

### 4.2. Antenatal Care

The antenatal care plan should include routine obstetric monitoring and surveillance for hemoglobinopathy [26]. The British Society for Hematology recommends that all patients with a hemoglobinopathy have the following at each visit: a clinical assessment, a blood pressure assessment, a urinalysis for symptomatic bacteriuria, and a full blood count at monthly intervals. Low-dose aspirin (75–150 mg daily) should be initiated at 12 weeks to reduce the risk of preeclampsia-related complications [4]. The British Society for Hematology advises that this should be offered to all women with hemoglobinopathy who are at risk of developing hypertensive disorders of pregnancy [4]. Maternal vitals should be monitored closely during pregnancy for VOCs, acute chest syndrome, and infection [2,9]. Serial fetal ultrasound scans are recommended, including a first-trimester ultrasound for dating, a second-trimester ultrasound for anatomy, and growth ultrasound scans every 4 weeks starting at 24 weeks into the pregnancy to check for evidence of IUGR [4]. Women with a prior history of poor pregnancy outcomes or with evidence of inadequate hepatic function should have increased antenatal fetal surveillance [4].

### 4.3. Transfusion

According to prophylactic transfusion studies, there appears to be an indication that the use of prophylactic transfusion was able to reduce perinatal death by seven times from baseline rates in 108 pregnancies of women with S-β-thalassemia, providing early evidence of this practice [27]. Currently, prophylactic transfusion is recommended for high-risk individuals, specifically those with a stroke history, women receiving chronic transfusions prior to pregnancy, and those who have frequent, severe VOCs or are transitioning from hydroxyurea. In a study of the HbSβ cohort, the mean hemoglobin was reported as 8.4 g/d [1].

Women without high-risk features who are to be transfused receive a conservative approach to transfusions. Transfusions are only recommended for acute complications such as severe anemia (Hb < 5–6 g/dL), acute chest syndrome, stroke, splenic sequestration, or preparation for cesarean delivery [4]. Regardless of the strategy employed, all units must be ABO-, Rh-, and Kell-matched, CMV-negative, and HbS-negative to reduce the risk of alloimmunization.

### 4.4. Iron Overload and Thromboprophylaxis

For patients with thalassemia major requiring blood transfusions with consequent iron overload, chelation therapy needs to be considered carefully. Deferasirox and deferiprone should not be used in pregnancy; deferoxamine can be continued into the second or third trimester if the benefits of treatment validate the risks of untreated iron overload [26]. Splenectomized patients and other women with risk factors require thromboprophylaxis, with low-dose aspirin routinely given antenatally and then switched to low-molecular-weight heparin (LMWH) from 32 weeks and/or postpartum [5].

### 4.5. Intrapartum and Postpartum

Mode of delivery is dictated by obstetric indications, though cesarean section rates are high across all cohorts; moreover, 83.9% was reported in Elenga et al.’s observational study [7], and median complication rates reached 77% in the Hb Sβ group [1]. According to Sunanda and Impana (2023), a vaginal birth at term can occur in women with sickle cell β-thalassemia with multidisciplinary care [3]. The use of routine cesarean sections must be avoided. The administration of hydration, oxygenation, and analgesics (including patient-controlled epidural analgesics when appropriate) during both labor and delivery will help to prevent sickling crises. As in the peri-partum period, hydration, electrolyte replacement, oxygen saturation, and effective pain control should be maintained [2]. According to the Society for Maternal-Fetal Medicine, women with a history of deep vein thrombosis or pulmonary embolism who undergo cesarean delivery should receive both mechanical thromboprophylaxis (starting before the operation and continuing until they are ambulatory) and pharmacologic prophylaxis, such as low-molecular-weight heparin, for 6 weeks after delivery [17].

## 5. Discussion

The current review discusses the evidence of pregnancy outcomes with HbSβ-thal and related SCD genotypes and additionally situates these findings against the supplementary comparator literature on SCT, a distinct and generally benign carrier state. The evidence shows that both the mother and newborn will experience significant morbidity during pregnancy. Advances in prenatal care and the use of multidisciplinary teams have improved maternal and fetal survival rates over the past few decades. However, women continue to be considered at high risk for complications (including obstetric, hematologic, and neonatal) throughout their entire pregnancy. By contrast, the SCT comparator literature more often showed outcomes close to the general obstetric population, with a smaller set of associations (preeclampsia, intrauterine fetal death) reported inconsistently and sometimes attenuated after adjustment for confounders [11].

A notable finding across all studies was an increased rate of preterm delivery, ranging from approximately 25% to 30% in large cohort studies and up to 48% in some recent US series [1,28]. SCD pregnancies have a significantly higher preterm birth rate than unaffected populations. In one report, SCD had a preterm birth rate of 28.5%, while the preterm birth rate for unaffected pregnancies was only 5.6%. An adjusted risk ratio of 2.62 was also reported in the literature for preterm births among patients with SCD [22]. These studies demonstrate that preterm birth continues to be an important obstetric risk in SCD pregnancies and is found across healthcare systems and geographical locations.

The data regarding fetal growth is equally concerning. The rate of LBW ranges from 56% to 62% in SCD [28]. In addition, the placental pathology behind fetal growth restriction has been increasingly characterized recently. A study identified that most of the placentas they looked at had evidence of maternal vascular malperfusion [1], and it was confirmed that mothers with low maternal hemoglobin during pregnancy (<8 g/dL) had significantly higher rates of fetal IUGR (47% vs. 14%, *p* = 0.0074) and that 79% of placentas from SCD patients had abnormalities of vascular malperfusion [24]. These mechanistic findings provide clear biological explanations for the observed growth restriction, including the roles of chronic hemolytic anemia, endothelial dysfunction, and recurrent vaso-occlusion in impairing uteroplacental blood flow, thereby resulting in suboptimal delivery of substrates to the developing fetus.

Neonates experience some level of morbidity in addition to growth measures. Apgar scores and neonatal intensive care unit admission (NICU) rates for newborns are two examples of morbidity sources that appear to have been well-established. These rates vary widely across populations and genotypes, ranging from 23% to 89% [29]. For genotypes with collected data, genotype appears to significantly affect the level of morbidity a neonate has experienced. According to a study, 66.7% (2 of 3) of HbSS newborns were admitted to a NICU, compared with 11.1% (1 of 9) of HbSC newborns, with a *p*-value of 0.016 [29]. This indicates that the severity of the underlying hemoglobinopathy directly correlates with the outcome of neonates born with such disorders. Further, the observation of neonates delivered from β-thalassemia minor pregnancies that suffered from jaundice and excessive weight loss, and/or were admitted to a NICU indicates that this group also poses a risk to neonates born from those pregnancies, hence warranting close monitoring in this population group [30,31].

There are a few limitations to note in this review. Most of the studies conducted are retrospective. This means they can be affected by selection bias or the quality of the data provided. Very few studies isolate HbSβ-thal as a distinct population; most data derive from larger SCD cohorts in which HbSβ-thal is a subgroup. Furthermore, the specific data concerning SCT and pregnancy remain limited, as there have mostly been retrospective studies performed with minimal evidence gathered from small populations, which does not provide sufficient insight into pregnancy risks associated with SCT. In addition, the SCT evidence base remains small and is dominated by retrospective cohorts drawn from single centers, so the trait comparison presented here should be regarded as indicative rather than definitive. A separate systematic review and meta-analysis restricted to β-thalassemia major and intermedia (429 participants; 684 pregnancies) similarly reported adverse pregnancy outcomes and disease-specific iron-status changes in that population, underscoring the value of genotype-specific analysis even outside the SCD spectrum [32]. Outcome definitions and participant classifications vary across studies, complicating comparison. Studies from low-resource settings, where SCD burden is often highest, were comparatively underrepresented and less detailed. Finally, of the fourteen included sources, three are secondary syntheses (two narrative reviews and a guideline incorporating a structured evidence review) rather than independent primary studies; where feasible, we have attributed specific figures to the primary literature synthesized within these sources.

The results of this study highlight, from a clinical perspective, the critical need for timely, multidisciplinary interventions for pregnant individuals with HbSβ-thal and other related hemoglobinopathies. Optimal management requires preconception counseling, early access to their prenatal provider, screening for infections, continuous monitoring of hemoglobin and blood pressure, and individually developed transfusion protocols. The potential of prophylactic transfusions to reduce preterm deliveries before 34 weeks (OR 0.05) requires confirmation through adequately powered randomized clinical trials [6].

Future research should focus on developing prospective, genotype-stratified studies examining outcomes specifically related to HbSβ-thal pregnancies; standardized definitions of neonatal morbidity utilized across different healthcare systems; improved access to multidisciplinary care in resource-limited settings; and dedicated prospective studies of SCT in pregnancy to resolve the inconsistent associations reported in the included literature.

## 6. Conclusions

In conclusion, women with HbSβ-thal still experience higher rates of morbidity during pregnancy, both for themselves and their fetuses. Despite continued advances in clinical care, women will continue to experience many complications related to sickle cell thalassemia during pregnancy, such as severe anemia, VOCs, preterm delivery, and LBW. Certain measures are suggested to significantly improve maternal and neonatal outcomes, including early disease identification, an MDT approach, close monitoring during pregnancy, and individualized treatment. By contrast, the supplementary evidence reviewed here suggests SCT carriers experience outcomes closer to the general obstetric population, though isolated associations (e.g., preeclampsia, intrauterine fetal death) warrant further prospective study. More prospective studies specific to HbSβ-thal are indicated to provide an evidence-based approach to pregnancy management.

## Data Availability

No new data were created or analyzed in this study. Data sharing is not applicable to this article.

## Abbreviations

AMSTAR 2	A MeaSurement Tool to Assess systematic Reviews (2nd version)
CARE	CAse REport guidelines
HbA	Hemoglobin A
HbAS	Hemoglobin AS genotype (sickle cell trait)
HbS	Hemoglobin S
HbSC	Hemoglobin SC disease
HbSS	Hemoglobin SS (homozygous sickle cell anemia)
HbSβ-thal	Hemoglobin S-beta-thalassemia (sickle cell-beta-thalassemia)
HbSβ^0^-thalassemia	Hemoglobin S-beta-zero-thalassemia
HbSβ^+^-thalassemia	Hemoglobin S-beta-plus-thalassemia
IUGR	Intrauterine growth restriction
LBW	Low birth weight
LMWH	Low-molecular-weight heparin
MDT	Multidisciplinary team
NICU	Neonatal intensive care unit
NOS	Newcastle–Ottawa Scale
PRISMA	Preferred Reporting Items for Systematic Reviews and Meta-Analyses
PROSPERO	International Prospective Register of Systematic Reviews
SANRA	Scale for the Assessment of Narrative Review Articles
SCD	Sickle cell disease
SCT	Sickle cell trait
SGA	Small for gestational age
VOC	Vaso-occlusive crisis
